# Genetic induction of hypometabolism by ablation of MC4R does not suppress ALS-like phenotypes in the G93A mutant SOD1 mouse model

**DOI:** 10.1038/s41598-017-13304-4

**Published:** 2017-10-13

**Authors:** Shachee Doshi, Preetika Gupta, Robert G. Kalb

**Affiliations:** 1 Division of Neurology, Department of Pediatrics, Children’s Hospital of Philadelphia, 3615 Civic Center Blvd, Philadelphia, PA 19104 USA; 2 Neuroscience Graduate Group, University of Pennsylvania, 140 John Morgan, 3620 Hamilton Walk, Philadelphia, PA 19104 USA; 3 Department of Neurology, University of Pennsylvania, 3400 Spruce St, Philadelphia, PA 19104 USA

## Abstract

Dysfunction and death of motor neurons leads to progressive paralysis in amyotrophic lateral sclerosis (ALS). Recent studies have reported organism-level metabolic dysfunction as a prominent but poorly understood feature of the disease. ALS patients are hypermetabolic with increased resting energy expenditure, but if and how hypermetabolism contributes to disease pathology is unknown. We asked if decreasing metabolism in the mutant superoxide dismutase 1 (SOD1) mouse model of ALS (G93A SOD1) would alter motor function and survival. To address this, we generated mice with the G93A SOD1 mutation that also lacked the melanocortin-4 receptor (MC4R). MC4R is a critical regulator of energy homeostasis and food intake in the hypothalamus. Loss of MC4R is known to induce hyperphagia and hypometabolism in mice. In the MC4R null background, G93A SOD1 mice become markedly hypometabolic, overweight and less active. Decreased metabolic rate, however, did not reverse any ALS-related disease phenotypes such as motor dysfunction or decreased lifespan. While hypermetabolism remains an intriguing target for intervention in ALS patients and disease models, our data indicate that the melanocortin system is not a good target for manipulation. Investigating other pathways may reveal optimal targets for addressing metabolic dysfunction in ALS.

## Introduction

Amyotrophic lateral sclerosis (ALS) is an adult-onset neurodegenerative disease characterized by loss of upper and lower motor neurons leading to paralysis and death^1–4^. Work from a variety of models implicates dysfunctional RNA metabolism, protein homeostasis, altered mitochondrial function and oxidative stress in disease pathophysiology^1,4,5^. In addition to cell-autonomous factors (i.e., the accumulation of toxic misfolded proteins in motor neurons), there is compelling evidence for cell nonautonomous processes contributing to disease progression, such as the participation of microglia and astrocytes^6,7^. At present these insights into ALS have not translated into drugs that substantively influence the course of disease.

Studies of patients with ALS have provided intriguing evidence for whole organism metabolic derangements. For example, glucose intolerance is present in 33% of sporadic ALS patients vs. 9.5% in controls^8^, hypermetabolism is present in 50–60% of ALS patients^9^ and dyslipidemia, measured by increased LDL/HDL ratio, is present in 45.4% of ALS patients vs 16.1% in controls^10^. ALS patients have lower body mass index (BMI) and lower lean body mass compared to healthy controls^11^, and many of these perturbations present early in disease and are progressive. There is an inverse correlation between premorbid BMI and risk of ALS; in overweight or obese individuals, the risk of ALS was reduced 30–40%^12^. Mouse models of ALS, based on known familial mutations such as SOD1, TDP43, FUS and C9ORF72, similarly show energetic abnormalities including mitochondrial dysfunction, decreased body weight and hypermetabolism^13–17^. ALS patients fed a high calorie, high carbohydrate diet showed fewer adverse events, delayed weight loss and longer lifespan than those fed a control diet^18^. Various types of high calorie diets have been shown to provide benefits to the mutant SOD1 mouse model of the disease as well^13,19,20^. Together, these observations provide evidence that organismal metabolism is likely to contribute to the pathogenesis of ALS.

One approach to investigating the contribution of hypermetabolism (an increase in resting energy expenditure) to ALS^9,21^ is to genetically decrease metabolic rate in mouse models of disease. For example, a previous study shows that placing the G93A SOD1 mice on a leptin deficient background (G93A;ob^+/−^) lowers organism level metabolism^22^. These mice gain more weight, become hypometabolic, have improved motor function as well as longer lifespan compared to G93A SOD1 mice alone. However, this study has several inherent limitations. For technical reasons, it was not possible to study G93A;ob/ob animals and thus the degree of hypometabolism achieved was rather modest. In addition, beyond its role in energy homeostasis (by controlling feeding and promoting satiety), leptin has myriad additional actions, including effects on neuronal development^23–25^. These limitations led us to consider if a different genetic manipulation that led to a more pronounced hypometabolic phenotype, and obviated the complexities of leptin deficiency, might lead to a greater improvement in the G93A SOD1 model.

**Figure 1:**
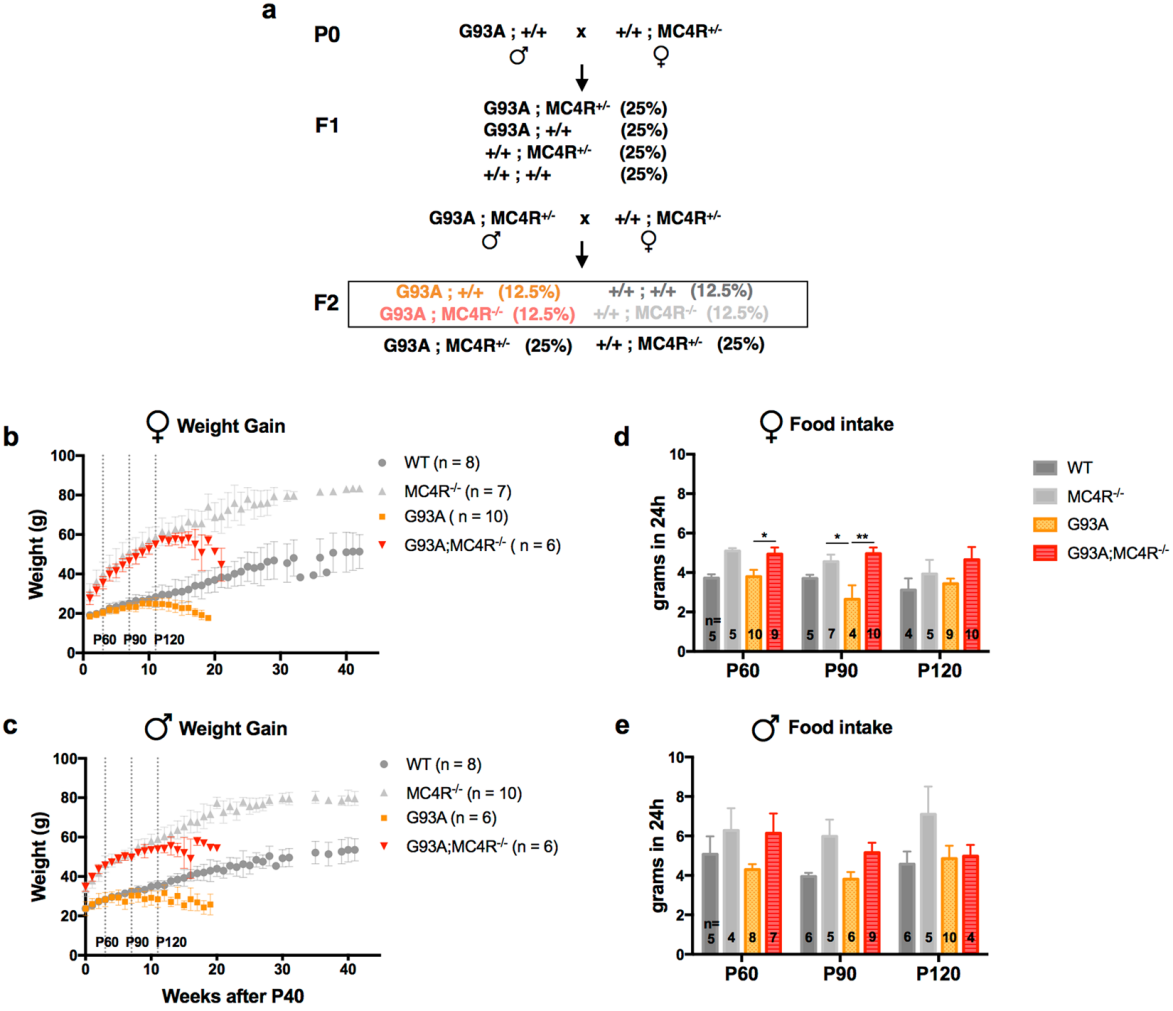
Breeding strategy, body weight and food intake in study mice. (**a**) Two step breeding strategy for generating study mice. Parentheses indicate the expected genotype frequencies of the progeny in each cross. The relevant animals used in this study are represented inside the box in the F2 generation. Body weight measurements in female (**b**) and male (**c**) WT, G93A, MC4R^−/−^ and G93A;MC4R^−/−^ mice, recorded weekly after P40. The vertical dashed lines represent P60, P90 and P120 when measurements of food intake, ambulatory activity, metabolic rate and motor function were made. Food intake measurements over a 24 h period at P60, P90 and P120 in female (**d**) and male (**e**) mice. Significance measured by one-way ANOVA among genotypes at each time point, followed by Tukey’s test for multiple comparisons: p < 0.05 (*), p < 0.01 (**), p,0.001 (***), p < 0.0001 (****).

To this end, we crossed G93A SOD1 mice, henceforth referred to as G93A, to those lacking the melanocortin-4 receptor (MC4R), henceforth referred to as MC4R^−/−^, generating double mutant G93A;MC4R^−/−^ mice (see methods and Fig. 1a for breeding strategy). We chose this gene since MC4R null mice are shown to be hypometabolic and hyperphagic^26,27^. Additionally, MC4R is a good candidate because of its central role in energy homeostasis^28^. It is expressed in the anterior bed nucleus of the stria terminalis, paraventricular nucleus of the hypothalamus and lateral hypothalamus and regulates food intake, metabolic rate and body weight. Mice lacking this receptor cannot promote energy expenditure, leading to hypometabolism, hyperphagia and weight gain. Our goal was to genetically induce hypometabolism in G93A mice by placing them in the MC4R null background, thus altering a central component of energy balance, and test the effect of this specific manipulation on metabolism, disease progression and survival.

## Results

### G93A mice lacking the melanocortin-4 receptor are obese, hyperphagic and lethargic

#### Body weight

Beginning at P40, WT mice (C57Bl/6) of both sexes gained weight over the subsequent 40 weeks (Fig. 1b,c). MC4R^−/−^ mice also gained weight over the same period, however, they were heavier than WT mice at every recorded time point. For example, at week 10 of measurements (P110), female and male WT mice weighed an average of 28.3 g and 34.9 g respectively, while MC4R^−/−^ mice weighed 58.6 g and 57.9 g respectively. G93A mice of both sexes also gained the same amount of weight as WT mice until about P103 (9–11 weeks after P40), after which they progressively lost weight until they were moribund. G93A;MC4R^−/−^ mice of both sexes gained the same amount of weight as MC4R^−/−^ mice until about P124 (11–12 weeks after P40), after which they progressively lost weight until they were moribund. At P110, female and male G93A mice weighed an average of 24.9 g and 29.2 g respectively, while G93A;MC4R^−/−^ mice weighed 55 g and 53.4 g respectively. These results indicate that, as previously reported^26,29^, MC4R^−/−^ mice gain significantly more weight compared to WT mice as they age, and G93A mice too gain significant weight when MC4R function is ablated, in comparison to WT and G93A animals.

#### Feeding

Next, we used CLAMS (see methods) to study food consumption in these mice over a 24 h period at P60, P90 and P120 (Fig. 1d,e). When all four genotypes (WT, G93A, MC4R^−/−^, G93A;MC4R^−/−^) were compared, group differences were found by one-way ANOVA for females (P60: *F*_(3,25)_ = 4.476, p = 0.012; P90: *F*_(3,21)_ = 5.924, p = 0.0043; P120: *F*_(3,21)_ = 1.56, p = 0.2287) and males (P60: *F*_(3,19)_ = 1.311, p = 0.2998; P90: *F*_(3,22)_ = 3.579, p = 0.0302; P120: *F*_(3,20)_ = 1.552, p = 0.232). Female WT mice consumed 3–4 g of food over 24 h at P60, P90 and P120, while male WT mice consumed 4–5 g of food at the same times. Female and male MC4R^−/−^ mice, in comparison, consumed more food than WT mice (4–5 g and 6–7 g respectively) at these time points but this did not achieve significance in *post hoc* analyses. Female and male G93A mice had similar amounts of food consumption compared to WT mice (3–4 g and 4–5 g respectively), and female and male G93A;MC4R^−/−^ mice had similar amounts of food consumption compared to MC4R^−/−^ mice (5 g and 5–6 g respectively). Importantly, female G93A;MC4R^−/−^ mice had significantly greater food consumption than G93A mice at P60 and P90 (*post hoc*, p < 0.05 at P60 and p < 0.01 at P90), but not at P120. Male G93A;MC4R^−/−^ mice consumed more food than G93A mice at all age points, but this did not reach statistical significance. These results demonstrate that WT and G93A mice have similar daily food consumption, and MC4R^−/−^ and G93A;MC4R^−/−^ mice have similar daily food consumption that is greater than WT and G93A mice.

#### Ambulatory activity

**Figure 2:**
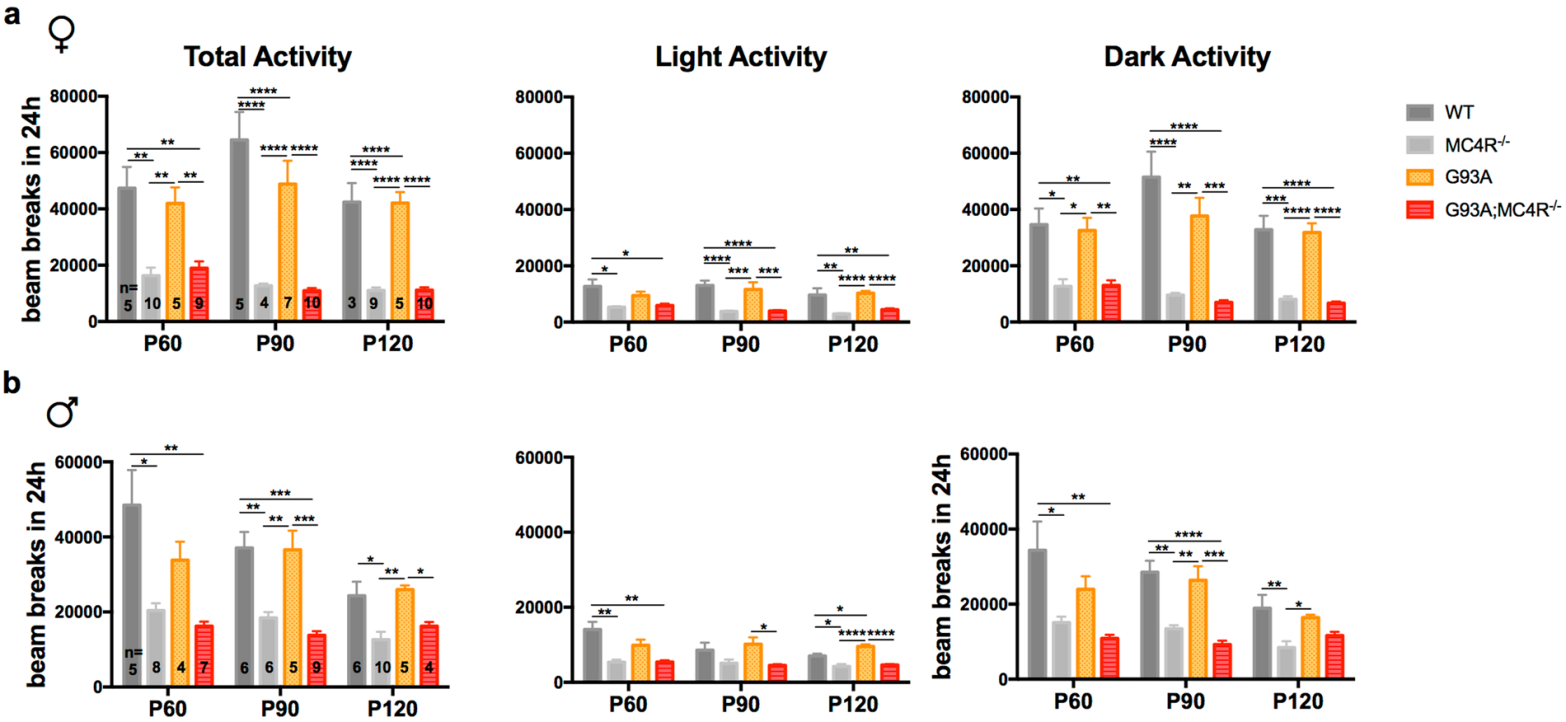
Ambulation in study mice. Ambulatory activity measured by beam breaks recorded at P60, P90 and P120 in female (**a**) and male (**b**) mice. Significance measured by one-way ANOVA among genotypes at each time point, followed by Tukey’s test for multiple comparisons: p < 0.05 (*), p < 0.01 (**), p,0.001 (***), p < 0.0001 (****).

We also used the CLAMS to look at ambulatory (non-grooming) activity of these mice over 24 h at P60, P90 and P120 (Fig. 2a,b). Three parameters were measured at each age: total activity over 24 h, activity during the 12 h light period and activity during the 12 h dark period. Group differences within the 4 genotypes (WT, G93A, MC4R^−/−^ and G93A;MC4R^−/−^) were determined using one-way ANOVA at each age, followed by *post hoc* analysis with Tukey’s test for multiple comparisons.

a) Total Activity. Group differences were found in total activity over 24 h at P60, P90 and P120 for females (P60: *F*_(3,26)_ = 9.546, p = 0.0002; P90: *F*_(3,24)_ = 36.55, p < 0.0001; P120: *F*_(3,23)_ = 34.36, p < 0.0001) and males (P60: *F*_(3,20)_ = 7.346, p = 0.017; P90: *F*_(3,22)_ = 15.31, p < 0.0001; P120: *F*_(3,21)_ = 8.289, p = 0.0008). MC4R^−/−^ mice of both sexes were significantly less active than WT mice at all three ages (*post hoc*, p < 0.05). G93A mice of both sexes were as active as WT mice at all three ages. G93A;MC4R^−/−^ mice of both sexes were as active as MC4R^−/−^ mice, and were significantly less active compared to G93A mice at all three ages for females and at P90 and P120 for males (*post hoc*, females p < 0.01, males p < 0.05).

b) Light Period Activity. Group differences were found in activity of the mice during the 12 h light period at P60, P90 and P120 in females (P60: *F*_(3,25)_ = 5.328, p = 0.0056; P90: *F*_(3,22)_ = 20.76, p < 0.0001; P120: *F*_(3,23)_ = 19.32, p < 0.0001) and males (P60: *F*_(3,20)_ = 7.939, p = 0.0011; P90: *F*_(3,22)_ = 4.413, p = 0.0142; P120: *F*_(3,21)_ = 19.51, p < 0.0001). MC4R^−/−^ mice of both sexes were significantly less active than WT mice at all three ages for females and at P60 and P120 for males (*post hoc*, p < 0.05). G93A mice of both sexes had similar activity compared to WT mice at all three ages. G93A;MC4R^−/−^ mice of both sexes were as active as MC4R^−/−^ mice, and were significantly less active compared to G93A mice at P90 and P120 (*post hoc*, females p < 0.001, males p < 0.05).

c) Dark Period Activity. Group differences were found in the activity of the mice during the 12 h dark period at P60, P90 and P120 in females (P60: *F*_(3,25)_ = 9, p = 0.0003; P90: *F*_(3,22)_ = 28.5, p < 0.0001; P120: *F*_(3,23)_ = 32.57, p < 0.0001) and males (P60: *F*_(3,20)_ = 6.436, p = 0.0032; P90: *F*_(3,22)_ = 17.46, p < 0.0001; P120: *F*_(3,20)_ = 6.302, p = 0.0035). MC4R^−/−^ mice of both sexes were significantly less active than WT mice at all three ages (*post hoc*, p < 0.05). G93A mice of both sexes had similar activity compared to WT mice at all three ages. G93A;MC4R^−/−^ mice of both sexes were as active as MC4R^−/−^ mice, and were significantly less active compared to G93A mice at P90 and P120 for females and at P90 for males (*post hoc*, females p < 0.01, males p = 0.0001).

These data indicate that WT and G93A mice have similar activity levels at the measured time points in both the light and the dark cycles. During the same periods, MC4R^−/−^ mice are significantly less active than WT mice and G93A;MC4R^−/−^ mice have similar activity levels as MC4R^−/−^ mice, significantly less than G93A mice alone.

In summary, G93A mice begin to lose weight compared to WT mice as they age. However, their food intake and activity levels are similar to WT mice at all recorded ages and even as they are losing weight, indicating a hypermetabolic phenotype. In all three measures - weight gain, food intake and activity level - G93A;MC4R^−/−^ mice are indistinguishable from MC4R^−/−^ mice. They are overweight, feed more and have greatly reduced activity compared to G93A mice, indicating that ablation of MC4R leads to unambiguous and significant phenotypic changes in G93A mice. Manipulating the activity of MC4R is thus a reasonable way to induce changes at the whole organism level in this mutant SOD1 background.

### G93A mice lacking the melanocortin-4 receptor are hypometabolic

**Figure 3:**
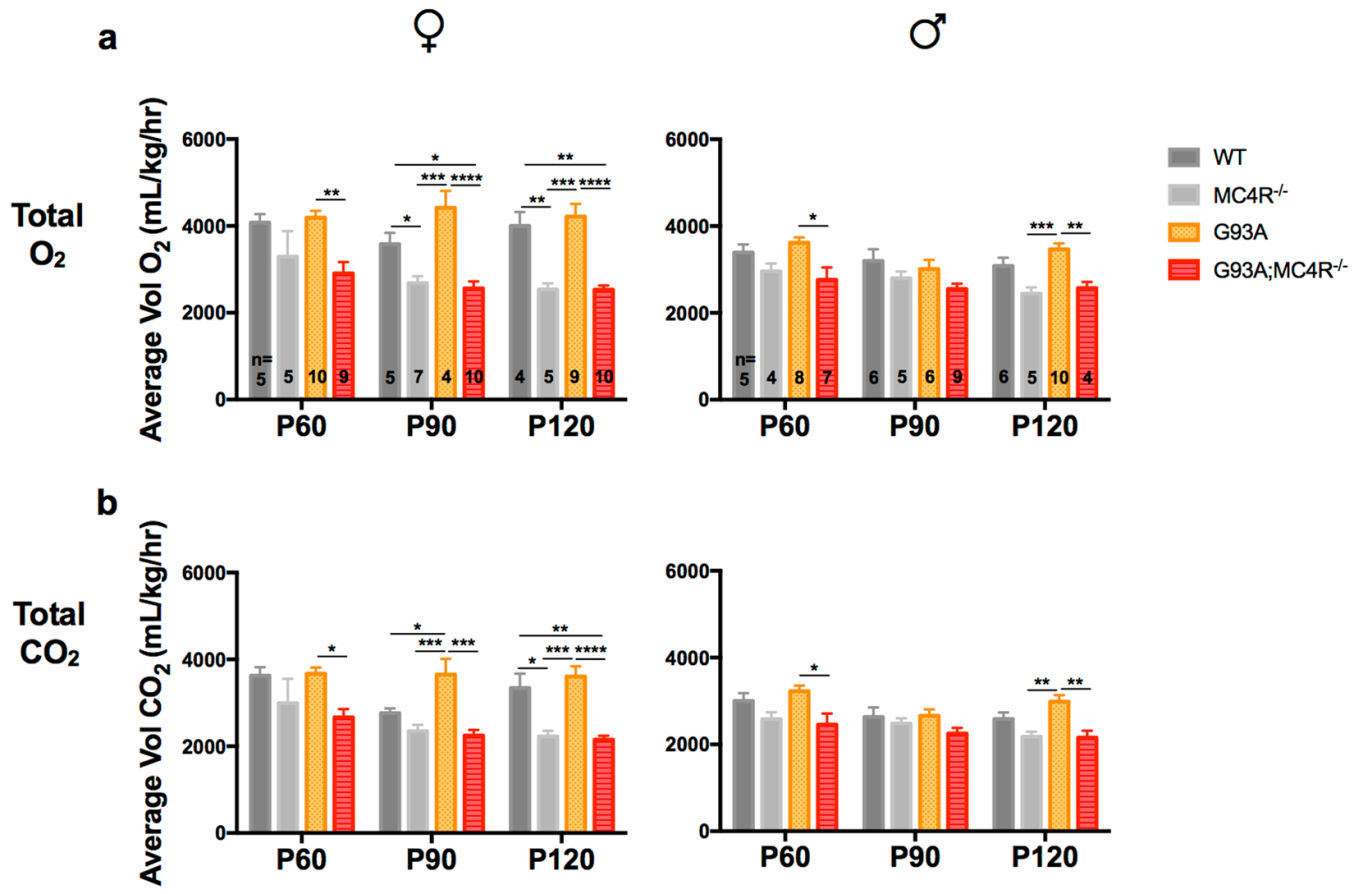
Oxygen consumption and carbon dioxide production over one day (24 h) in study mice. Total O_2_ consumption (**a**) and CO_2_ production (**b**) in female and male WT, G93A, MC4R^−/−^ and G93A;MC4R^−/−^ mice at P60, P90 and P120 measured in mL, per hour, per kg mass of the animal over 24 hours of recording, including light and dark cycles. Significance measured by one-way ANOVA among genotypes at each time point, followed by Tukey’s test for multiple comparisons: p < 0.05 (*), p < 0.01 (**), p,0.001 (***), p < 0.0001 (****).

**Figure 4:**
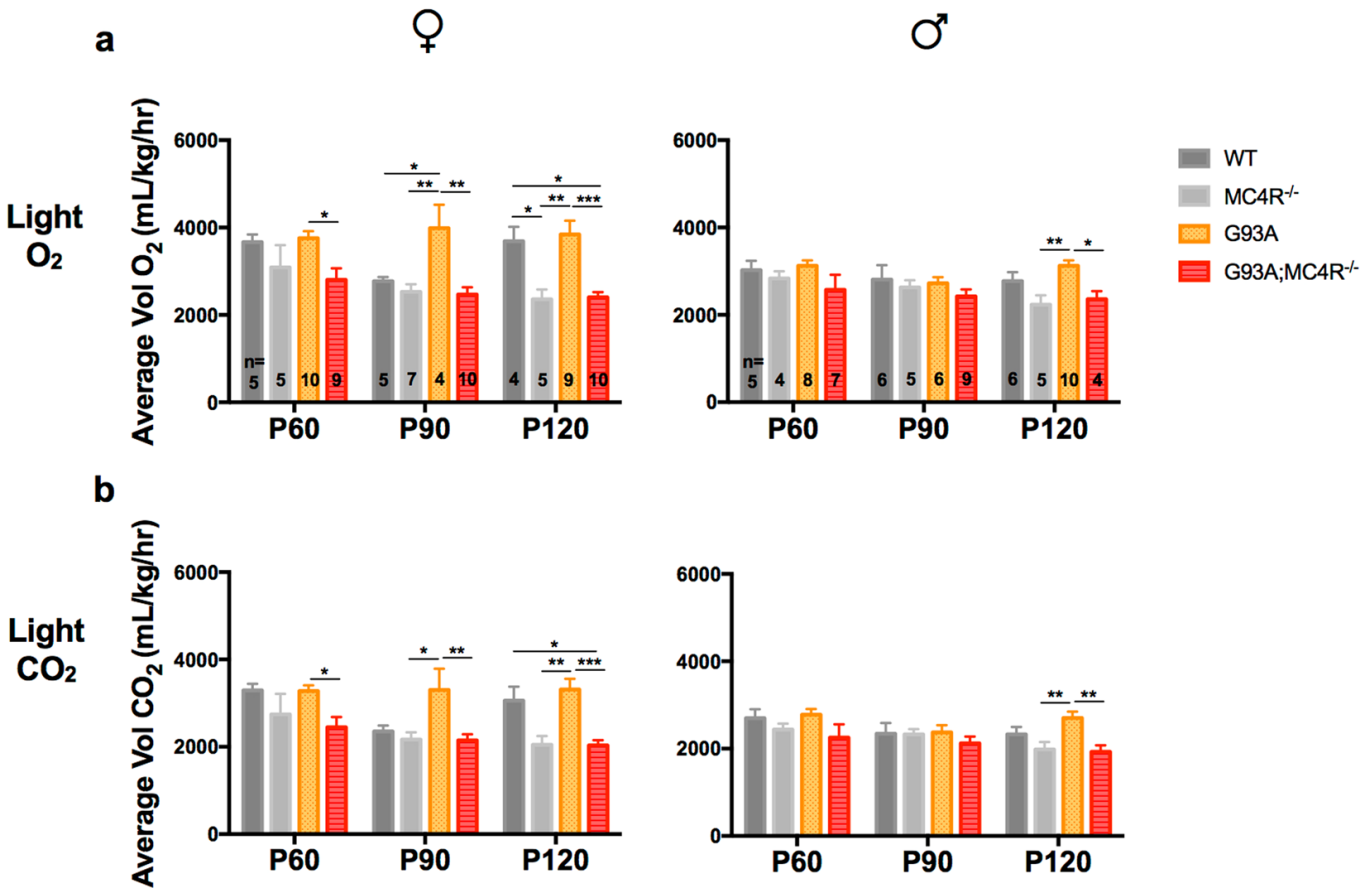
Oxygen consumption and carbon dioxide production during the 12 h light cycle in study mice. Light cycle O_2_ consumption (**a**) and CO_2_ production (**b**) in female and male WT, G93A, MC4R^−/−^ and G93A;MC4R^−/−^ mice at P60, P90 and P120 measured in mL, per hour, per kg mass of the animal over 12 hours of recording during the light cycle of the day. Significance measured by one-way ANOVA among genotypes at each time point, followed by Tukey’s test for multiple comparisons: p < 0.05 (*), p < 0.01 (**), p,0.001 (***).

**Figure 5:**
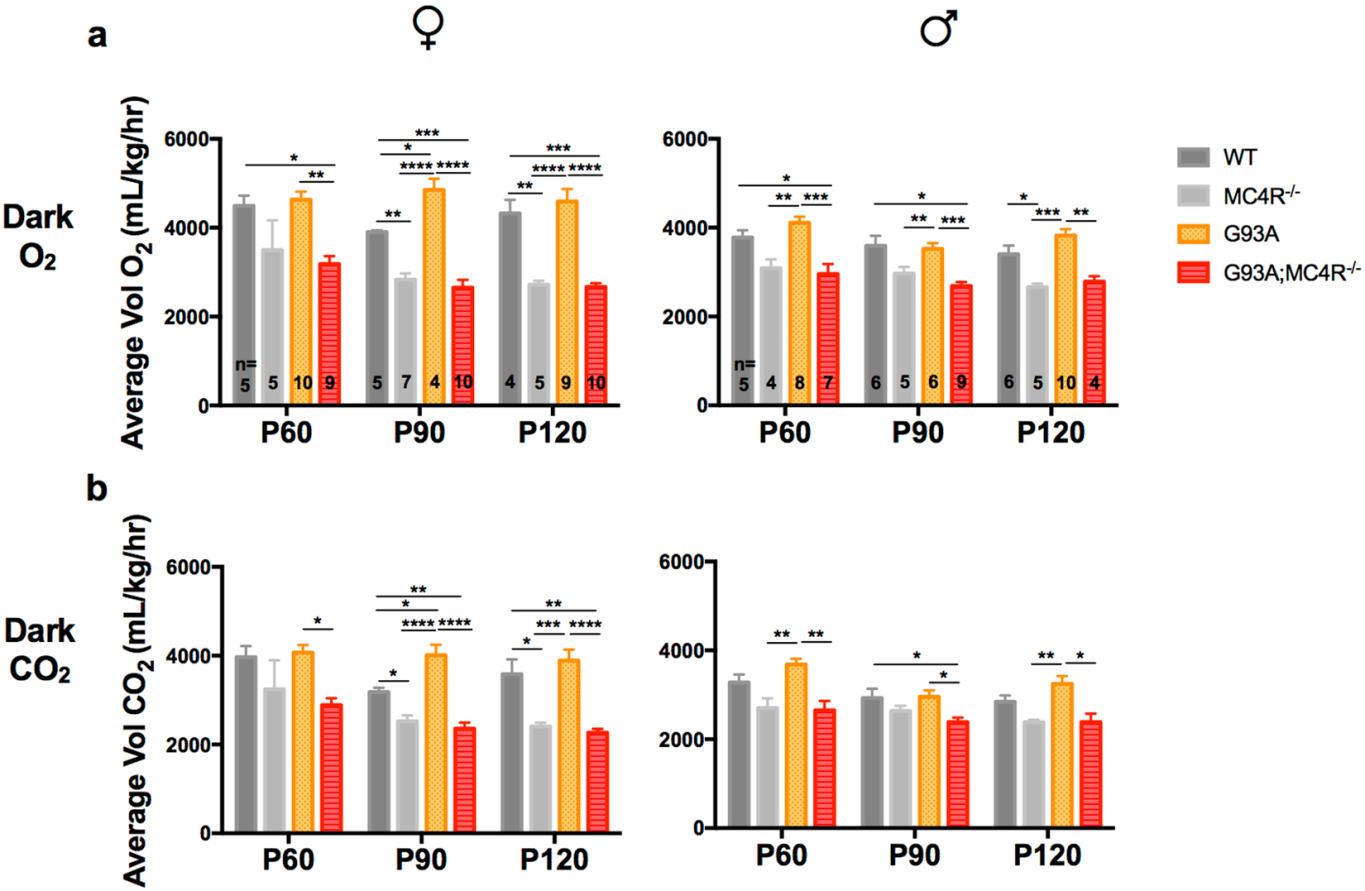
Oxygen consumption and carbon dioxide production during the 12 h dark cycle in study mice. Dark cycle O_2_ consumption (**a**) and CO_2_ production (**b**) in female and male WT, G93A, MC4R^−/−^ and G93A;MC4R^−/−^ mice at P60, P90 and P120 measured in mL, per hour, per kg mass of the animal over 12 hours of recording during the dark cycle of the day. Significance measured by one-way ANOVA among genotypes at each time point, followed by Tukey’s test for multiple comparisons: p < 0.05 (*), p < 0.01 (**), p,0.001 (***), p < 0.0001 (****).

In order to determine metabolic flux in test mice over time, we measured average total oxygen consumption and carbon dioxide production per hour, per kg over a 24 h period at the three different ages (P60, P90 and P120) using indirect calorimetry by the CLAMS (Fig. 3a,b). We also looked at the O_2_ consumption and CO_2_ production during the 12 h light period (Fig. 4a,b) and 12 h dark period (Fig. 5a,b) within the 24 h recording. Group differences between the genotypes (WT, G93A, MC4R^−/−^ and G93A;MC4R^−/−^) were determined using one-way ANOVA at each age, followed by *post hoc* analysis with Tukey’s test for multiple comparisons.

#### Total O_2_ consumption

Group differences in O_2_ consumption over 24 h were found at P60, P90 and P120 for females (P60: *F*_(3,25)_ = 5.259, p = 0.0060; P90: *F*_(3,22)_ = 13.61, p < 0.0001; P120: *F*_(3,24)_ = 17.07, p < 0.0001) and males (P60: *F*_(3,20)_ = 3.3916, p = 0.0238; P90: *F*_(3,22)_ = 2.434, p = 0.0919; P120: *F*_(3,21)_ = 9.607, p = 0.0003). Female MC4R^−/−^ mice had significantly less O_2_ consumption than WT mice at P90 and P120 (*post hoc*, p < 0.05), while male MC4R^−/−^ mice had decreased O_2_ consumption compared to WT mice at all three ages but this did not achieve significance. Female G93A mice had significantly more O_2_ consumption than WT mice at P90 (*post hoc*, p < 0.05), whereas male G93A mice had increased O_2_ consumption compared to WT mice at P60 and P120 but this did not achieve significance. G93A;MC4R^−/−^ mice of both sexes were similar compared to MC4R^−/−^ mice, and had significantly less O_2_ consumption compared to G93A mice at all three ages for females and at P60 and P120 for males (*post hoc*, females p < 0.01, males p < 0.05) (Fig. 3a).

#### Total CO_2_ production

Group differences in CO_2_ production over 24 h were found at P60, P90 and P120 for females (P60: *F*_(3,25)_ = 4.258, p = 0.0147; P90: *F*_(3,22)_ = 10.67, p = 0.0002; P120: *F*_(3,24)_ = 15.85, p < 0.0001) and males (P60: *F*_(3,20)_ = 3.852, p = 0.0252; P90: *F*_(3,22)_ = 1.681, p = 0.2002; P120: *F*_(3,21)_ = 6.699, p = 0.0024). Female MC4R^−/−^ mice had significantly less CO_2_ production than WT mice at P120 (*post hoc*, p < 0.05), while male MC4R^−/−^ mice had decreased CO_2_ production than WT mice at all three ages but this was not statistically significant. Female G93A mice had significantly greater CO_2_ production than WT mice at P90 (*post hoc*, p < 0.05), whereas male G93A mice had increased CO_2_ production compared to WT mice at P60 and P90 but this was not statistically significant. G93A;MC4R^−/−^ mice of both sexes were similar compared to MC4R^−/−^ mice, but had significantly less CO_2_ production compared to G93A mice at all three ages for females and at P60 and P120 for males (*post hoc*, p < 0.05 for females and males) (Fig. 3b).

#### Light Period O_2_ consumption

Group differences in oxygen consumption during the12h light period were found at P60, P90 and P120 for females (P60: *F*_(3,25)_ = 3.271, p = 0.0378; P90: *F*_(3,22)_ = 7.161, p = 0.0016; P120: *F*_(3,24)_ = 10.16, p = 0.0002) and males (P60: *F*_(3,20)_ = 1.16, p = 0.3498; P90: *F*_(3,22)_ = 0.747, p = 0.5357; P120: *F*_(3,21)_ = 5.867, p = 0.0045). Female MC4R^−/−^ mice had significantly less O_2_ consumption than WT mice at P120 (*post hoc*, p < 0.05), while males had decreased O_2_ consumption compared to WT mice at P60 and P120, but this was not significant. Female G93A mice had significantly greater O_2_ consumption than WT mice at P90 (*post hoc*, p < 0.05), whereas male G93A mice had increased O_2_ consumption compared to WT mice at P120, but this was not significant. G93A;MC4R^−/−^ mice of both sexes were similar compared to MC4R^−/−^ mice, but had significantly less O_2_ consumption compared to G93A mice at all three ages for females and at P120 for males (*post hoc*, females p < 0.05, males p < 0.05) (Fig. 4a).

#### Light Period CO_2_ production

Group differences in CO_2_ production during the 12 h light period were found at P60, P90 and P120 for females (P60: *F*_(3,25)_ = 3.337, p = 0.0354; P90: *F*_(3,22)_ = 5.136, p = 0.0076; P120: *F*_(3,24)_ = 10.79, p = 0.0001) and males (P60: *F*_(3,20)_ = 1.326, p = 0.2939; P90: *F*_(3,22)_ = 0.4866, p = 0.6951; P120: *F*_(3,21)_ = 5.126, p = 0.0081). MC4R^−/−^ mice of both sexes had decreased CO_2_ production than WT mice at P60 and P120 but this was not statistically significant. G93A mice had increased CO_2_ production compared to WT mice at P90 and P120 in females and P120 in males but this was not statistically significant. G93A;MC4R^−/−^ mice of both sexes were similar compared to MC4R^−/−^ mice, but had significantly less CO_2_ production compared to G93A mice at all three ages for females and at P120 for males (*post hoc*, p < 0.05 for females and males) (Fig. 4b).

#### Dark Period O_2_ consumption

Mice of all genotypes had increased O_2_ consumption in the dark compared the light period, consistent with their increased nocturnal activity. Group differences in O_2_ consumption during the 12 h dark period were found at P60, P90 and P120 for females (P60: *F*_(3,25)_ = 6.772, p = 0.0017; P90: *F*_(3,22)_ = 29.86, p < 0.0001; P120: *F*_(3,24)_ = 24.78, p < 0.0001) and males (P60: *F*_(3,20)_ = 9.646, p = 0.0004; P90: *F*_(3,22)_ = 9.709, p = 0.0003; P120: *F*_(3,21)_ = 11.98, p < 0.0001). MC4R^−/−^ mice of both sexes had significantly less O_2_ consumption than WT mice, at P90 and P120 for females and at P120 for males (*post hoc*, females p < 0.01, males p < 0.05). Female G93A mice had significantly greater O_2_ consumption than WT mice at P90 (*post hoc*, p < 0.05), whereas male G93A mice had increased O_2_ consumption compared to WT mice at P60 and P120, but this was not statistically significant. G93A;MC4R^−/−^ mice of both sexes were similar compared to MC4R^−/−^ mice, and had significantly less O_2_ consumption compared to G93A mice at all three ages (*post hoc*, p < 0.01) (Fig. 5a).

#### Dark Period CO_2_ production

Similar to O_2_ consumption, mice of all genotypes had increased CO_2_ production in the dark compared the light period, consistent with their increased nocturnal activity. Group differences in CO_2_ production during the 12 h dark period were found at P60, P90 and P120 for females (P60: *F*_(3,25)_ = 4.646, p = 0103; P90: *F*_(3,22)_ = 20.56, p < 0.0001; P120: *F*_(3,24)_ = 19.56, p < 0.0001) and males (P60: *F*_(3,20)_ = 8.061, p = 0.0010; P90: *F*_(3,22)_ = 3.994, p = 0.0206; P120: *F*_(3,21)_ = 6.135, p = 0.0037). Female MC4R^−/−^ mice had significantly less CO_2_ production than WT mice at P90 and P120 (*post hoc*, p < 0.05), whereas male MC4R^−/−^ mice had decreased CO_2_ production compared to WT mice at all ages but this was not statistically significant. Female G93A mice had significantly increased CO_2_ production compared to WT mice at P90 (*post hoc*, p < 0.05), while male G93A mice had increased CO_2_ production compared to WT mice at P60 and P120 but this was not statistically significant. G93A;MC4R^−/−^ mice of both sexes were similar compared to MC4R^−/−^ mice, but had significantly less CO_2_ production compared to G93A mice at all three ages (*post hoc*, p < 0.05 for females and males) (Fig. 5b).

Together, these data from indirect calorimetry suggest that compared to WT animals, G93A mice display a moderately higher metabolic rate and that MC4R^−/−^ mice are hypometabolic. Interestingly, both the G93A hypermetabolism and the MC4R^−/−^ hypometabolism is more striking in females than in males, and during the dark cycle. When G93A mice are placed in the MC4R^−/−^ background, their metabolic profile is similar to MC4R^−/−^ mice alone for both males and females, i.e. G93A;MC4R^−/−^ mice are significantly more hypometabolic compared to G93A mice in both dark and light periods. This suggests that MC4R ablation, in addition to affecting weight, food intake and activity, can also rescue the hypermetabolic phenotype in this mutant SOD1 model of ALS.

### G93A mice lacking the melanocortin-4 receptor do not have improved motor function

Motor function of G93A mice deteriorates over time^30^. We asked if induction of hypometabolism blunted this age dependent phenotype. To this end, we measured forelimb and hindlimb grip strength in male and female study mice at P60, P90 and P120.

#### Forelimb Strength

**Figure 6:**
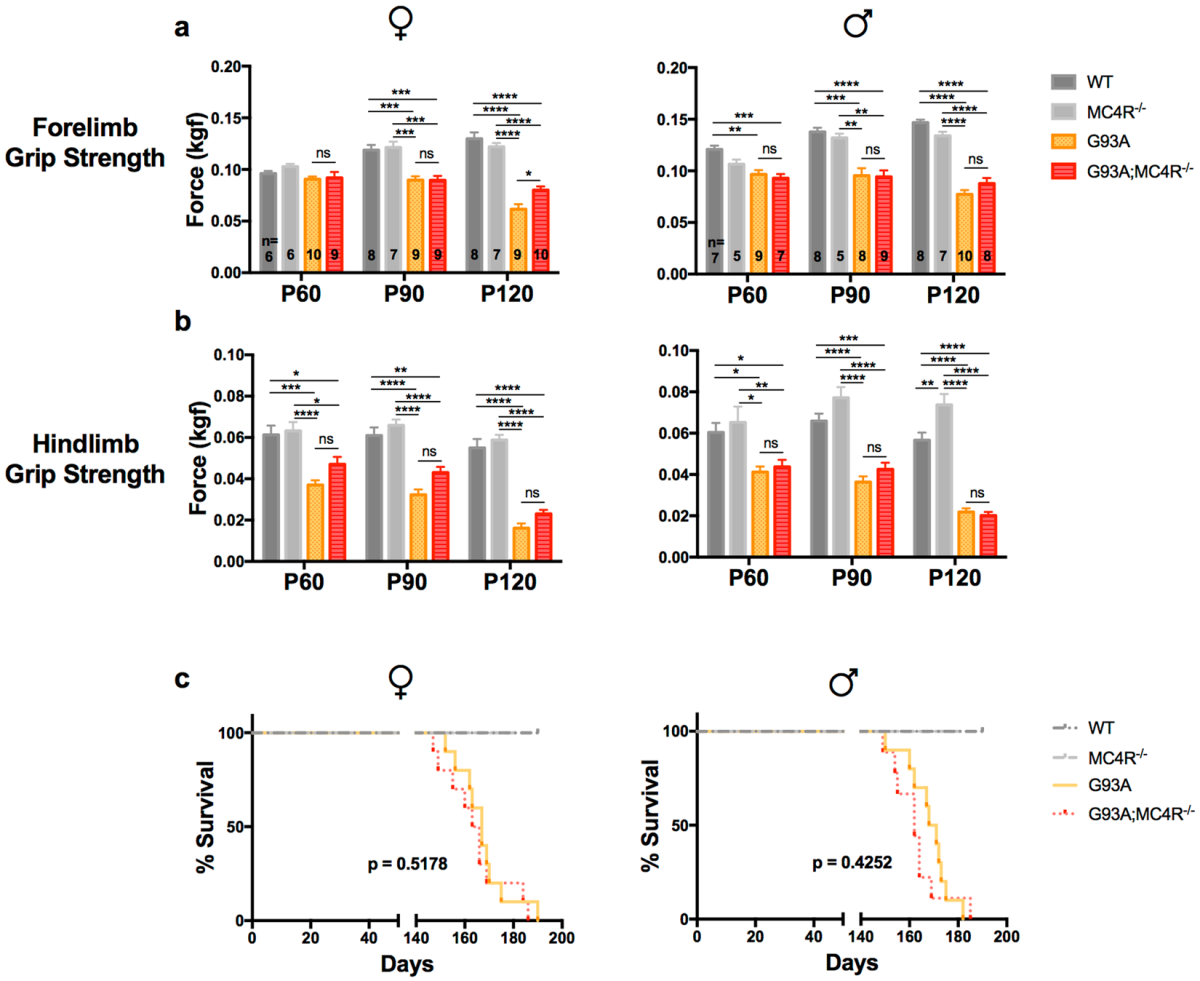
Motor function and survival of study mice. Forelimb (**a**) and hindlimb (**b**) grip strength of female and male WT, G93A, MC4R^−/−^ and G93A;MC4R^−/−^ mice measured at P60, P90 and P120. Significance measured by one-way ANOVA among genotypes at each time point, followed by Tukey’s test for multiple comparisons: p < 0.05 (*), p < 0.01 (**), p,0.001 (***), p < 0.0001 (****). Kaplan-Meier survival plots (**c**) of female and male study mice. Significance was calculated using the log-rank (Mantel-Cox) test for survival curve comparison between G93A and G93A;MC4R^−/−^ mice, and was set to p < 0.05.

Group differences in forelimb grip strength among genotypes (WT, MC4R^−/−^, G93A, G93A;MC4R^−/−^) were found using one-way ANOVA at each age point for females (P60: *F*_(3,27)_ = 1.724, p = 0.1856; P90: *F*_(3,29)_ = 12.77, p < 0.0001; P120: *F*_(3,30)_ = 50.14, p < 0.0001) and males (P60: *F*_(3,24)_ = 9.174, p = 0.0003; P90: *F*_(3,26)_ = 15.69, p < 0.0001; P120: *F*_(3,29)_ = 66.43, p < 0.0001) (Fig. 6a).

MC4R^−/−^ mice of both sexes had similar forelimb grip strength compared to WT mice at all three ages. G93A mice of both sexes had significantly decreased forelimb grip strength compared to WT mice, at P90 and P120 for females and at all three ages for males (*post hoc*, females p < 0.001, males p < 0.01). G93A;MC4R^−/−^ mice of both sexes had significantly decreased forelimb grip strength than MC4R^−/−^ mice at P90 and P120 (*post hoc*, females p < 0.001, males p < 0.01), and had similar forelimb grip strength compared to G93A mice at all three ages. The only exception was that female G93A;MC4R^−/−^ mice had significantly improved grip strength compared to G93A mice at P120 (*post hoc*, p < 0.05).

#### Hindlimb Strength

Group differences in hindlimb grip strength among genotypes (WT, MC4R^−/−^, G93A, G93A;MC4R^−/−^) were found using one-way ANOVA at each age point for females (P60: *F*_(3,27)_ = 12.66, p < 0.0001; P90: *F*_(3,29)_ = 25.69, p < 0.0001; P120: *F*_(3,30)_ = 57.73, p < 0.0001) and males (P60: *F*_(3,24)_ = 7.494, p = 0.0010; P90: *F*_(3,26)_ = 26.7, p < 0.0001; P120: *F*_(3,29)_ = 68.35, p < 0.0001) (Fig. 6b).

MC4R^−/−^ mice of both sexes had similar hindlimb grip strength compared to WT mice at all three ages, with one exception: male MC4R^−/−^ mice had significantly increased hindlimb strength compared to WT mice at P120 (*post hoc*, p = 0.0051). G93A mice of both sexes had progressively worsening hindlimb grip strength with age, and this was significantly decreased compared to WT mice at all three ages (*post hoc*, females p < 0.001, males p < 0.05). This difference was larger as the animals aged, at P90 and P120. G93A;MC4R^−/−^ mice of both sexes had significantly decreased hindlimb grip strength than MC4R^−/−^ mice all three ages (*post hoc*, p < 0.05). Although female G93A;MC4R^−/−^ mice had increased hindlimb grip strength compared to G93A mice at all ages, this did not achieve significance. Male G93A;MC4R^−/−^ mice had similar hindlimb grip strength compared to G93A mice at all three ages.

These experiments show that G93A mice have significantly less forelimb and hindlimb grip strength compared to WT mice, indicating progressively impaired motor function. Importantly, although G93A;MC4R^−/−^ mice are similar to MC4R^−/−^ mice in their hypometabolic phenotype, they are significantly worse than MC4R^−/−^ mice in grip strength. In fact, male G93A;MC4R^−/−^ mice are indistinguishable from G93A mice in grip strength. In females, there is significantly improved forelimb strength at P120 in G93A;MC4R^−/−^ mice compared with G93A mice, and there is a consistent trend in improved hindlimb strength at all time points. Therefore, MC4R ablation has no effect on the motor function of male G93A mice and has a modest effect in female G93A mice.

### G93A mice lacking the melanocortin-4 receptor do not have extended lifespan

Finally we looked at the lifespan of our mice. WT and MC4R^−/−^ mice had similar longevity and no animals of these genotypes died within 1 year of birth. In agreement with published work, we found male and female G93A mice had a severely shortened lifespan compared to WT mice^31^, with a median survival of 169.5 days and 167 days respectively (Fig. 6c). There was no significant difference in the lifespan of G93A;MC4R^−/−^ mice compared to G93A mice. Median survival in these mice was 162 days and 164.5 days for males and females respectively (Fig. 6c). Although the double mutant G93A;MC4R^−/−^ mice reach the pre-determined euthanasia criteria at the same time as G93A mice, they continue to have significantly higher body weight than the G93A mice at end stage (Fig. 1b,c). Together, these data suggest that ablation of melanocortin-4 receptor does not significantly prolong the life span of the G93A mouse.

### The leptin-MC4R pathway is altered in mice lacking the melanocortin-4 receptor

In addition to their role in controlling food intake and organismal metabolism, peptide hormones that impinge upon the MC4R signaling pathway can display beneficial neuronal activities. In its canonical role in regulating metabolism, leptin acts on two populations of neurons in the arcuate nucleus of the hypothalamus: the proopiomelanocortin (POMC)-producing neurons and the agouti-related peptide (AgRP)-producing neurons^28^. The POMC derived α-melanocyte stimulating hormone (α-MSH) is an agonist and AgRP is an antagonist of the MC4R. When calories are replete, α-MSH activates MC4R to promote satiety, feeding suppression, and weight loss by energy expenditure. When calories are depleted AgRP antagonizes MC4R to promote energy consumption and blunt energy expenditure. With regard to beneficial neuronal activities, α-MSH can promote cognitive recovery in a mouse model of Alzheimer’s disease^32^ and demonstrates general anti-inflammatory properties^33,34^. Similarly, higher circulating leptin is associated with decreased risk of Alzheimer’s disease and protection against cognitive decline^35^. Due to their varied actions, we asked if the expression of serum leptin and α-MSH might be altered in G93A and MC4R^−/−^ animals in order to clarify the role of these peptides in our experiments.

**Figure 7:**
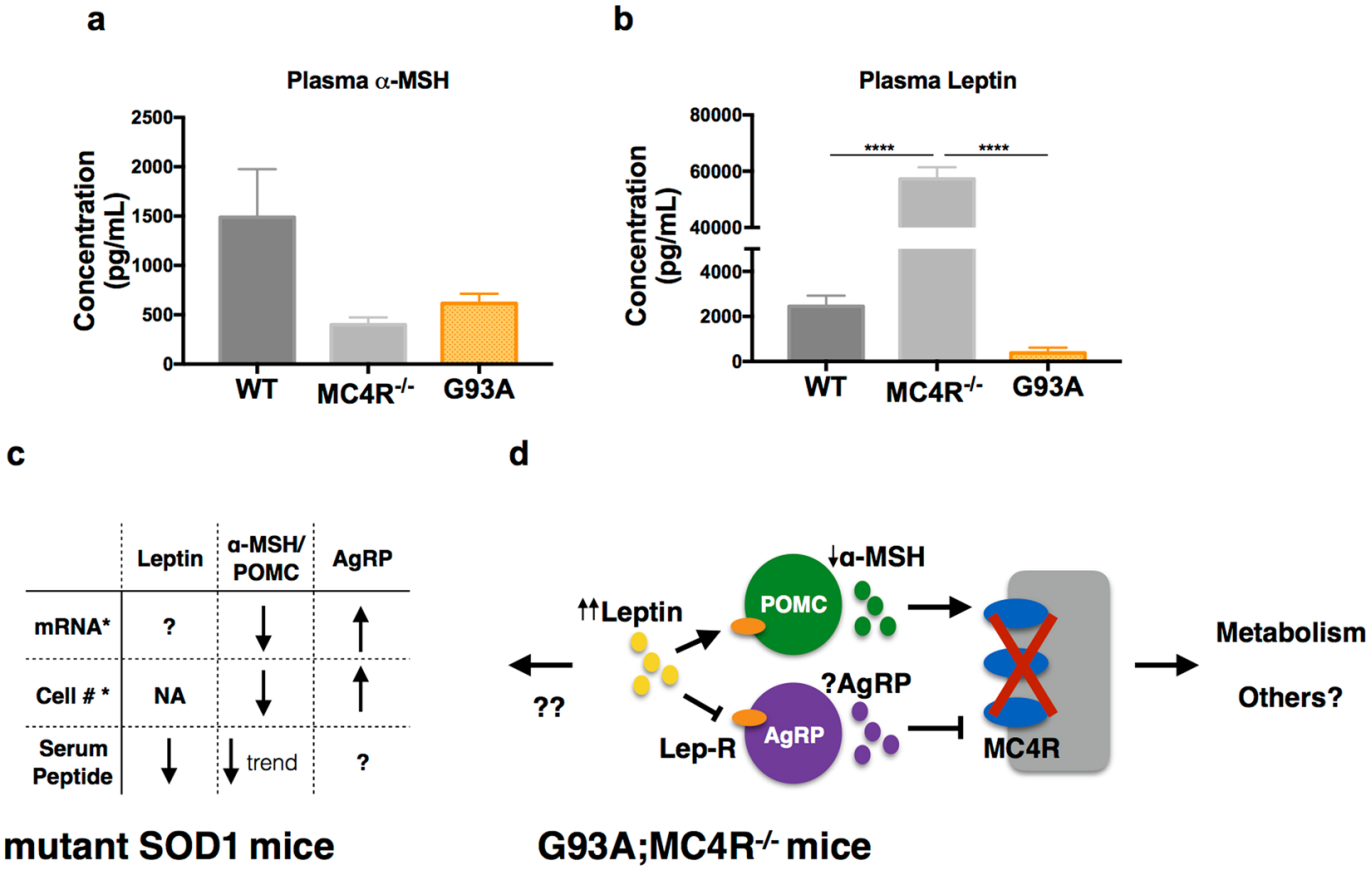
The leptin-MC4R pathway in G93A SOD1 mice. Plasma α-MSH (**a**) and leptin (**b**) concentrations in adult male WT, MC4R^−/−^ mice (n = 3 for each genotype). Significance measured by one-way ANOVA among genotypes, followed by Tukey’s test for multiple comparisons: p < 0.0001 (****). (**c**) Summary of alterations in leptin, α-MSH/POMC cells and AgRP/AgRP cells in *G86R SOD1 mice (Vercruysse *et al*.^44^) and G93A SOD1 mice. (**d**) Speculative model of the leptin-MC4R pathway in G93A;MC4R^−/−^ mice based on data from MC4R^−/−^ mice.

We measured plasma concentrations of leptin and α-MSH in adult male WT, MC4R^−/−^ and G93A mice. α-MSH levels were decreased in both MC4R^−/−^ and G93A mice compared to WT (Fig. 7a), but this did not achieve significance (p = 0.08 for MC4R^−/−^ and p = 0.16 for G93A). Leptin levels are moderately decreased in G93A mice (Fig. 7b) and markedly increased in MC4R^−/−^ mice compared to WT animals (p < 0.0001), consistent with the increased body weight and adiposity of these animals (Fig. 1). As seen in previous work^13,22^, leptin levels were decreased in G93A mice compared to WT mice. The modest difference in α-MSH levels among the genotypes may indicate relatively little contribution of the extra-metabolic effects of this hormone on neuronal health. The markedly elevated leptin levels in the absence of MC4R may indicate a healthful compensatory hormonal response, although if so, it does not improve motor function or extend life of the G93A mice. In light of the benefits of haploinsufficiency of leptin demonstrated by Lim *et al*.^22^, it is conceivable that increased leptin in the absence of MC4R actually mitigates any potential benefits of hypometabolism.

## Discussion

An increase in resting energy expenditure, or hypermetabolism, is a distinct organism-level feature of ALS patients and mouse models^9,13,36,37^. Our goal was to generate an ALS model mouse that was hypometabolic, and test the effect of this manipulation on key features of disease. In order to induce hypometabolism, we ablated MC4R function because it is a central regulator of energy metabolism. We successfully generated and studied mice that are null for MC4R and bear the ALS-linked G93A mutation in the SOD1 gene. We make two principal observations. First, the G93A;MC4R^−/−^ animals are markedly hypometabolic as reported by weight gain and indirect calorimetry. Thus the G93A mutation does not impede the central regulation of metabolism via MC4R. Second, despite evoking hypometabolism in the G93A animals, the most important measures of disease (e.g., motor function and longevity) are unaffected. This argues that genetic blunting of the hypermetabolic phenotype by manipulation of MC4R is an ineffective intervention for slowing disease progression.

### Different mechanisms of inducing hypometabolism in ALS

Prior studies in the G93A mouse model have shown that hypermetabolism does contribute to disease progression, and we highlight two of them here. In 2004, Dupuis *et al*. studied male mice in two different ALS-related mutant SOD1 mouse models, G86R and G93A^13^. They found that in both models, mice had lower body weight and increased energy expenditure by indirect calorimetry compared to age matched littermate controls. When the G86R mice were fed a high fat diet consisting of regular chow supplemented with 21% butterfat and 0.15% cholesterol, they gained significantly more weight and had increased fat deposits than those fed regular chow alone. The high fat diet was associated with slowed motor neuron loss, slowed muscle denervation and a 20% improvement in mean lifespan. In 2014, Lim *et al*. showed that the G93A mice were hypermetabolic and decreasing leptin levels (by placing them in a heterozgous ob^+/−^ background), led to hypometabolism and weight gain in these mice^22^. Leptin deficiency also led to improved hindlimb grip strength and led to a female-specific increase in median lifespan. These studies demonstrate that ameliorating the hypermetabolic phenotype, either by diet or by a specific genetic manipulation, can blunt the progression of ALS-like phenotypes in mutant SOD1 models of ALS.

What does the difference between our current results with MC4R and prior work by Dupuis *et al*. and Lim *et al*. tell us about the contribution of hypometabolism to ALS? To address this, it is worth considering leptin and its interaction with MC4R. Leptin is a circulating hormone that plays a prominent role in controlling food intake and satiety^28,38^. It is released by adipocytes and acts on specific neurons in the arcuate nucleus of the hypothalamus. These first order neurons then act on second order neurons in the lateral hypothalamus and the paraventricular nucleus of the hypothalamus. MC4R is expressed on the second order neurons that receive opposing inputs based on upstream leptin signaling. When calories are replete, leptin-mediated MC4R activation promotes satiety and energy expenditure. When calories are depleted, circulating leptin is reduced and MC4R is inhibited leading to food consumption and suppression of energy expenditure. On the surface, the leptin deficient G93A;ob^+/−^ mice Lim *et al*. studied and the G93A;MC4R^−/−^ mice we studied are similar; they are both heavier than G93A mice and are hypometabolic. Despite this similarity, it is surprising that ablating MC4R, leptin’s downstream target, does not confer the same benefits on motor function as reduced leptin signaling does. One possibility is that the beneficial effects of reduced leptin signaling acts via MC4R independent pathways. Indeed, leptin receptors are expressed in extrahypothalamic neurons in the midbrain and the cerebellum and in peripheral tissue such as the liver^39–42^ although the function of leptin signaling in extrahypothalamic tissues is not well understood. The reduction in circulating leptin that Lim *et al*. achieved in the ob^+/−^ background may point to important effects of leptin not captured by MC4R ablation. In light of this, we show here that mice lacking MC4R have a 20–30 fold increase in circulating leptin (Fig. 7b) and perhaps this mitigates any potential benefit of hypometabolism induced by loss of MC4R.

### The hypothalamic melanocortin pathway in ALS

Recent work indicates intrinsic abnormalities in the hypothalamus in ALS. Gorges and colleagues find significant atrophy of the hypothalamus in sporadic and familial ALS patients^43^. Atrophy presents in presymptomatic stages in familial mutation carriers and is more severe in patients with lower BMI. This suggests that changes in the hypothalamus precede changes in metabolism in ALS patients. In a different study, Vercruysse and colleagues looked specifically at the hypothalamic melanocortin system in ALS patients and the G86R mutant SOD1 mouse model^44^. In the G86R SOD1 mice, there are significantly fewer first order, MC4R-activating (POMC-expressing) neurons and significantly more first order, MC4R-inhibiting (AgRP containing) neurons compared to WT mice. These differences were observed at pre-symptomatic stages and suggest that signaling in the melanocortin system is downregulated in this ALS model. We too find changes in the MC4R signaling pathway (e.g., leptin and α-MSH are decreased in G93A mice, Fig. 7a and b), although we studied the G93A SOD1 model. To test the idea that evoking a hypometabolic state would be beneficial, Vercruysse *et al*. administered an inhibitor of the melanocortin system, pioglitazone. This did not lead to weight gain in a clinical trial of ALS patients and did not lead to increased food intake by the G86R SOD1 mice. Vercruysse *et al*. attributed the lack of efficacy of pioglitazone to the intrinsic abnormalities in melanocortin system. On the other hand we show that deletion of MC4R in the G93A mouse can clearly evoke hypometabolism, regardless of the state of the melanocortin system in ALS. Our data suggest that even if Vercruysse *et al*. could pharmacologically inhibit melanocortin system and evoke a hypometabolic state, it would not lead to a meaningful effect on weakness in these models.

Together, this study, Vercruysse *et al*.^47^, Dupuis *et al*.^13^ and Lim *et al*.^22^ show that leptin levels are lower in mutant SOD1 mice, there are fewer POMC neurons, more AgRP neurons and lower trending α-MSH (Fig. 7c). Given that loss of MC4R leads to a large increase in leptin levels, we speculate that contrary to G93A mice, leptin levels are also upregulated in the G93A;MC4R^−/−^ mice while α-MSH levels are lowered (Fig. 7d). If this is in fact the case, our study also suggests that upregulating leptin in mutant SOD1 mice may not be beneficial in altering disease course.

### Sex, metabolism and ALS

Differences between males and females may contribute to the effectiveness of metabolic manipulations in ALS. We find a small but significant female-specific improvement in forelimb grip strength at P120 in G93A;MC4R^−/−^ mice. In the leptin study, Lim *et al*. showed that the increase in median lifespan in G93A;ob^+/−^ mice compared to G93A mice was female-specific. Dupuis *et al*. only used male mice in their high-fat diet experiments, but a recent study showed that a diet with low unsaturated fatty acids led to female-specific decrease in median lifespan and a worsening of disease progression in G93A mice^45^. Similarly, metabolic manipulations are shown to affect male and female animals differently in other ALS studies^22,46–48^.

In our study, male MC4R^−/−^ mice have 22% lower O_2_ consumption and 16% lower CO_2_ production at P120 compared to WT mice in the active, dark period. In contrast, female MC4R^−/−^ mice at the same age and time of day have 37% lower O_2_ consumption and 33% lower CO_2_ production than WT mice. This clearly shows a stronger MC4R mediated hypometabolic phenotype in females. The results are similar in the G93A background. Male G93A;MC4R^−/−^ double mutant mice have 18% lower O_2_ consumption and 16% lower CO_2_ production than WT mice at P120 in the dark. In contrast, female G93A;MC4R^−/−^ mice have 38% lower O_2_ consumption and 37% lower CO_2_ production than WT mice at the same age and phase of day. This indicates that even in the G93A background, loss of MC4R has a stronger hypometabolic effect in females, which coincides with improved forelimb strength in females at the same age. Lim *et al*. found that G93A females had similar circulating leptin as WT mice while G93A males had decreased circulating leptin compared to WT^22^. Higher baseline leptin in female mice may contribute to their differential response to metabolic manipulations in the Lim *et al*. study and this study. These observations highlight sex-specific perturbations in metabolism and ALS.

There are well-documented metabolic differences between males and females, including effects of sex hormones such as estrogen and testosterone^49,50^, differences in fat storage^51^ and food intake^52^, to name a few. As highlighted above with leptin, metabolic differences are also present between males and females in ALS. In addition, there are sex-specific differences in ALS presentation and progression. The overall incidence of ALS is higher in males than in females, and spinal cord onset is more likely in males while bulbar onset is more likely in females^53^. In G93A mice, females have later onset and longer survival in different genetic backgrounds^54^, females have preferentially upregulated proteasomal activity^55^ and loss of certain genes like PGC-1α accelerates disease progression in males but not females^46^. The complex interaction of metabolic differences with pathophysiology and the differences in metabolism and disease progression in ALS are just beginning to be studied. It is therefore important to address male and female subjects differently in any ALS study, and especially those that perturb metabolism.

### The G93A SOD1 mouse model of ALS

Different features of ALS in patients may not be recapitulated in every model of the disease. Here, we chose the G93A SOD1 mouse since it is the most widely studied model of ALS. These mice mimic many of the clinical features of the disease in patients, including hypermetabolism. Unfortunately, there is no systematic study of the level of hypermetabolism specifically in patients with SOD1 mutations. Hence, it is not known whether the hypermetabolism in the SOD1 mouse model mimics patient symptoms. However, a small study showed that patients with familial ALS have a higher incidence of hypermetabolism than those with sporadic ALS^9^, and SOD1 mutations comprise up to 20% of familial ALS cases. Detailed analysis of the metabolic abnormalities in different SOD1 mutations both in patients and in mouse models will help in designing and interpreting studies of the contribution of metabolism in ALS.

Further, the mutant SOD1 strain we used expresses high levels of the human G93A SOD1 transgene, leading to a 4-fold increase in SOD1 activity^31^. Compared to other mouse models with different mutations in SOD1, this line has earlier symptom onset (13–17 weeks) and shorter lifespan (17–26 weeks). In contrast, the G85R mutant SOD1 mouse model has a later symptom onset (35–43 weeks) and longer lifespan (37–45 weeks)^56^. It is conceivable that the more aggressive presentation of ALS-like symptoms in the G93A mouse blunts the potential therapeutic benefit of hypometabolism. Dupuis *et al*. described a hypermetabolic phenotype in G93A and G86R mutant SOD1 mice, both of which have earlier onset of symptoms and short lifespans^13^, but it is unknown if the G85R mice have a hypermetabolic phenotype. It is thus important to determine the hypermetabolic phenotype in the G85R SOD1 model, and if it exists, to test the effect of lowering organismal metabolism in that model.

### Molecular mechanisms of hypermetabolism in mutant SOD1-linked ALS

Despite decades of effort, the mechanism by which mutant SOD1 causes motor neuron death remains unclear. However, accumulating data suggests that soluble, and not aggregated, misfolded mutant SOD1 could lead to cellular damage by promiscuous interactions with different molecular pathways, including mitochondrial dysfunction and reactive oxygen species production^57^. Damaged mitochondria can thus affect cellular metabolism, and by extension organismal metabolism. It is plausible that misfolded, soluble mutant SOD1 in the G93A mice is not affected by ablating MC4R and is therefore unable to change disease progression in these mice. Indeed, it is not known if metabolic changes are caused by disease-linked mutations or if metabolism is distinct from underlying mutations given the prevalence of aberrant metabolism in sporadic ALS patients.

## Conclusion

While experiments have shown that weight gain, increasing BMI, and decreasing energy expenditure are associated with lower risk for developing ALS^12,58,59^, not all genetic manipulations for inducing weight gain and altering metabolism are equal in their effectiveness. The findings presented here add to the growing understanding of how energetic dysfunction is coupled to ALS. It is critical to understand the metabolic profile in ALS thoroughly, and to detail exactly how different dietary and genetic changes influence metabolism in ALS in a sex-specific way in order to design appropriate therapeutic interventions.

## Methods

### Mouse strains and husbandry

Male hemizygous G93A mutant SOD1 mice (G93A^+/*−*^) on the C57BL/6 background (Strain #004435, Jackson Laboratories, Bar Harbor, ME), referred to as G93A, were crossed with female heterozygous loxTB MC4R mice (MC4R^+/−^) in the C57BL/6 background^29^ (Strain #006414, Jackson Laboratories, Bar Harbor, ME). The G93A mice carry one copy of the human G93A mutant SOD1 transgene, and the MC4R^+/−^ mice contain a transcriptional block cassette before the MC4R start codon. From the F1 progeny, G93A;MC4R^+/−^ males were crossed with MC4R^+/−^ females. From the F2 generation, the following groups were used for study purposes: WT (+/+), G93A, MC4R^−/−^, G93A;MC4R^−/−^ (Fig. 1a). Both males and females from each group were used for all study parameters. Genotypes were determined by PCR using tail snip DNA using the following primers – MC4R: GCAGTACAGCGAGTCTCAGG (wild type forward), CTCCCACAGGCTTATGACACC (wild type reverse), GTGCAAGTGCAGGTGCCAG (mutant), and SOD1: CTAGGCCACAGAATTGAAAGATCT (genomic forward), GTAGGTGGAAATTCTAGCATCATCC (genomic reverse), CATCAGCCCTAATCCATCTGA (transgene forward), CGCGACTAACAATCAAAGTGA (transgene reverse).

Mice were housed at 22 °C with a 12-hour light dark cycle. They were fed standard diet (23% protein, 22% fat, 55% carbohydrates). Animals were weighed once a week at the same time of day starting at P40 for at least 20 weeks. Mice without the G93A mutation were measured for over 40 weeks. All animal protocols were approved by the Institutional Animal Care and Use Committee (IACUC) at the Children’s Hospital of Philadelphia, and animals were treated in accordance with the National Institutes of Health Guide for the Care and Use of Laboratory Animals.

### Study Design

Mice of both sexes with four genotypes (WT, G93A, MC4R^−/−^, G93A;MC4R^−/−^) were studied. The following parameters were measured at P60, P90 and P120: oxygen consumption, carbon dioxide production, food consumption, ambulatory activity, forelimb strength and hindlimb strength. P60 is shown to be presymptomatic in G93A mice, while P90 is symptomatic and P120 is close to end stage^60^. We chose these time points in order to monitor progression of ALS-related and metabolic phenotypes as animals age. Animals were sacrificed when they were unable to right themselves within 30 seconds from being supine, and this was recorded as day of death for survival measurements.

### Weight gain, food consumption, activity and metabolic measurements

Food consumption, ambulatory activity and metabolic measurements were made at the appropriate time points (P60, P90 and P120) using the Comprehensive Laboratory Animal Monitoring System (CLAMS) (Columbus Instruments, Columbus OH). Animals were weighed and placed in individual chambers with food and water for 48 hours. Food consumption was measured in grams by recording the decrease in weight of the pre-measured food provided in the chambers. Ambulatory activity was measured in arbitrary units by the number of infrared beam breaks in the x-y plane of the cage every minute, thus ruling out grooming-related activity in the z-axis. Oxygen consumption and carbon dioxide production were measured by indirect calorimetry approximately every 10 minutes and reported in milliliters per hour, normalized by the body weight of the animal (mL/hr/kg). Animals were allowed to acclimate to the chambers for the first 24 hours, and only the data from the final 24 hours was used for analysis.

### Motor Output

Grip strength measurements were made at the same time of day at appropriate time points for the fore and hind paws using a digital grip strength meter (Columbus Instruments, Columbus, OH). For fore grip measurements, animals were allowed to grip a bar with their fore paws while being held by their tails, and gently pulled back until they let go of the bar. The meter recorded the force (in kilogram-force or kgf) with which they hold on to the bar as they are pulled back. Similarly, hind grip strength was measured as mice gripped a bar with their hind paws. For each animal, one set of trials comprised 3–5 consecutive trials, and each set was repeated three times, with approximately 3 minutes between sets for the animal to rest. The average strength for the 12–15 trials was reported for both the fore and the hind paws at each time point.

### Plasma leptin and α-MSH measurements

Mice were anesthetized using carbon dioxide and cardiac extraction was immediately performed to collect blood from adult WT, G93A and MC4R^−/−^ male mice. Blood was spun down at 2000 g for 20 minutes and supernatant plasma was frozen at −80 C until further use. Plasma concentrations of leptin and α-MSH were determined by the Radioimmunoassay and Biomarkers Core facility at the University of Pennsylvania using standard ELISA (Leptin: Cat # 22-LEPMS-E01 from ALPCO, Salem NH; α-MSH: Cat # MBS2516107 from MyBioSource, San Diego CA).

### Statistics

Data were analyzed using Prism (GraphPad Software, La Jolla, CA). Unless otherwise noted, significant differences within groups were determined using one-way ANOVA followed by Tukey’s test for multiple comparisons. Survival curves were analyzed using the log-rank (Mantel-Cox) test for significance. For all tests, the significance threshold was set to p < 0.05.

### Data Availability

The datasets generated during and/or analyzed during the current study are available from the corresponding author on reasonable request.
